# A Robust Cross-Platform Solution With the Sense2Quit System to Enhance Smoking Gesture Recognition: Model Development and Validation Study

**DOI:** 10.2196/67186

**Published:** 2025-05-20

**Authors:** Anarghya Das, Juntao Feng, Maeve Brin, Patricia Cioe, Rebecca Schnall, Ming-Chun Huang, Wenyao Xu

**Affiliations:** 1 Department of Computer Science & Engineering University at Buffalo, The State University of New York Buffalo, NY United States; 2 School of Nursing Columbia University New York, NY United States; 3 School of Public Health Brown University Providence, RI United States; 4 Columbia University School of Public Health Columbia University New York, NY United States; 5 Department of Data and Computational Science Duke Kunshan University Jiangsu China

**Keywords:** smoking cessation, confounding gestures, mobile health, wearable technology, real-time monitoring, people living with HIV, smoking gestures, smartwatch, mobile application, convolutional neural networks, vaping, e-cigarette, mobile phone

## Abstract

**Background:**

Smoking is a leading cause of preventable death, and people with HIV have higher smoking rates and are more likely to experience smoking-related health issues. The Sense2Quit study introduces innovative advancements in smoking cessation technology by developing a comprehensive mobile app that integrates with smartwatches to provide real-time interventions for people with HIV attempting to quit smoking.

**Objective:**

We aim to develop an accurate smoking cessation app that uses everyday smartwatches and an artificial intelligence model to enhance the recognition of smoking gestures by effectively addressing confounding hand gestures that mimic smoking, thereby reducing false positives. The app ensures seamless usability across Android (Open Handset Alliance [led by Google]) and iOS platforms, with optimized communication and synchronization between devices for real-time monitoring.

**Methods:**

This study introduces the confounding resilient smoking model, specifically trained to distinguish smoking gestures from similar hand-to-mouth activities used by the Sense2Quit system. By incorporating confounding gestures into the model’s training process, the system achieves high accuracy while maintaining efficiency on mobile devices. To validate the model, 30 participants, all people with HIV who smoked cigarettes, were recruited. Participants wore smartwatches on their wrists and performed various hand-to-mouth activities, including smoking and other gestures such as eating and drinking. Each participant spent 15 to 30 minutes completing the tasks, with each gesture lasting 5 seconds. The app was developed using the Flutter framework to ensure seamless functionality across Android and iOS platforms, with robust synchronization between the smartwatch and smartphone for real-time monitoring.

**Results:**

The confounding resilient smoking model achieved an impressive *F*_1_-score of 97.52% in detecting smoking gestures, outperforming state-of-the-art models by distinguishing smoking from 15 other daily hand-to-mouth activities, including eating, drinking, and yawning. Its robustness and adaptability were further confirmed through leave-one-subject-out evaluation, demonstrating consistent reliability and generalizability across diverse individuals. The cross-platform app, developed using Flutter (Google), demonstrated consistent performance across Android and iOS devices, with only a 0.02-point difference in user experience ratings between the platforms (iOS 4.52 and Android 4.5). The app’s continuous synchronization ensures accurate, real-time tracking of smoking behaviors, enhancing the system’s overall utility for smoking cessation.

**Conclusions:**

Sense2Quit represents a significant advancement in smoking cessation technology. It delivers timely, just-in-time interventions through innovations in cross-platform communication optimization and the effective recognition of confounding hand gestures. These improvements enhance the accuracy and accessibility of real-time smoking detection, making Sense2Quit a valuable tool for supporting long-term cessation efforts among people with HIV trying to quit smoking.

**International Registered Report Identifier (IRRID):**

RR2-10.2196/49558

## Introduction

### Background

The global health burden of tobacco use remains a formidable challenge for public health initiatives, with smoking-related illnesses claiming approximately 6 million lives worldwide annually [1]. Despite decades of efforts to reduce smoking rates through various interventions, including public education campaigns, taxation policies, and cessation programs, tobacco use persists as the leading preventable cause of morbidity and premature mortality worldwide [2]. The issue of smoking is prevalent and challenging to address among people with HIV. Approximately 50% of the 1 million people with HIV living in the United States smoke cigarettes, which is about 4 times higher than the prevalence observed in the general US adult population [3]. This highlights the urgent need for innovative, technology-driven smoking cessation interventions and real-time monitoring of smoking behaviors. Recent research has shown an increasing focus on developing smartphone apps for this purpose [4]. These apps offer various features such as SMS reminders, progress tracking, and peer support [5]. Concurrently, the proliferation of wearable technology has opened new avenues for continuous health monitoring and behavior modification. Integrating wearable devices into smoking cessation programs holds considerable potential for enhancing intervention efficacy and enabling real-time monitoring of smoking behaviors [6,7]. Smartwatches and smartphones with sensors can detect smoking events in real time, offering opportunities for timely interventions. Wearable technology, particularly smartwatches equipped with accelerometers and gyroscopes, provides a promising avenue for automatic smoking detection and intervention [8,9]. For instance, the SmokeBeat app [10], which leverages wearable sensors to identify smoking gestures, has shown promising results in smoking reduction by alerting users to their smoking episodes. Leveraging advancements in machine learning, these devices can analyze sensor data to accurately identify smoking gestures [11-13] and even presmoking activities, potentially offering real-time support for individuals attempting to quit [14,15]. However, despite the demonstrated feasibility and promising results in controlled settings, several critical challenges hinder these technologies’ widespread adoption and effectiveness. One major limitation is the lack of focus on high-risk populations with unique needs, such as people living with HIV. These groups, despite exhibiting distinct behavioral patterns and facing higher smoking rates, remain underrepresented mainly in current research efforts. Furthermore, there is a noticeable gap between technological innovation and evidence-based practice. While numerous studies explore the technical feasibility of smoking detection, few demonstrate how these systems can be effectively integrated into real-world settings while adhering to established health care guidelines. Most available mobile apps lack input from health care professionals and a strong theoretical framework [16], raising concerns about their efficacy and practical impact. This disconnect is compounded by the scarcity of research showcasing end-to-end solutions that combine automatic smoking detection with the unobtrusive and user-friendly nature of everyday devices such as smartwatches and smartphones. Finally, technical challenges persist, including maintaining reliable connectivity between wearable devices and smartphones [9] and the trade-off between detection accuracy and resource consumption [17]. Using multiple sensors or higher sampling rates can improve accuracy but increases battery drain, hindering usability [18]. This challenge is particularly relevant when considering real-world deployment [19], where the performance of smoking detection models often degrades compared to controlled laboratory settings. One key factor contributing to this decline is the presence of confounding gestures, which interfere with the model’s ability to detect smoking-specific movements accurately [20]. Confounding gestures are hand movements that closely mimic smoking behaviors, including eating, drinking, yawning, or applying chapstick. These gestures pose considerable challenges due to their repetitive hand-to-mouth motions, often causing false positive detections, undermining user trust, and reducing intervention systems’ effectiveness. Moreover, distinguishing smoking from less repetitive but visually similar activities presents additional difficulty, further complicating accurate gesture recognition.

**Figure 1:**
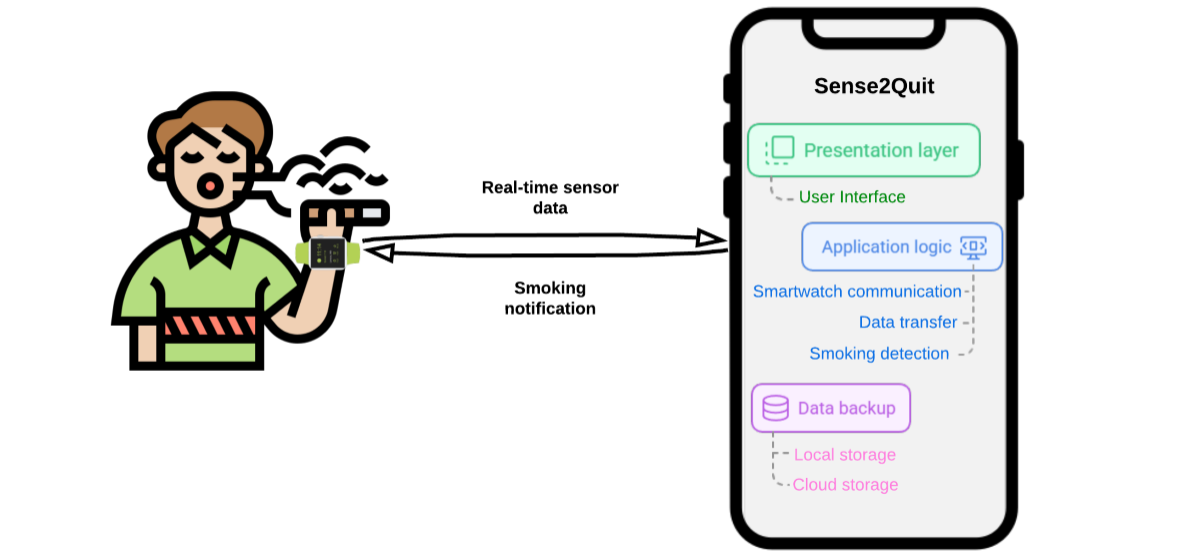
Overview of the Sense2Quit smartphone and smartwatch system architecture.

Our Sense2Quit study addresses critical gaps in current smoking cessation apps by following a rigorous, theoretically guided development framework and closely collaborating with health care professionals. This study aims to develop mHealth (mobile health) technology to support tobacco cessation in people with HIV. The mobile app supports people with HIV quitting smoking by using smartwatches for behavioral assessment and delivering just-in-time interventions. The primary research question driving this study is: can integrating confounding gesture data into the training of a smoking gesture recognition model significantly enhance its accuracy and real-time applicability in a high-risk population, specifically people living with HIV? We hypothesize that explicitly incorporating confounding gesture data into the model training process will significantly reduce false positives and negatives, thus improving overall accuracy and real-world effectiveness. Importantly, this approach does not require additional sensor inputs or increased computational complexity; instead, it uses targeted training enhancements to address the issue of confounding gestures, ensuring that the model remains efficient and practical for real-time deployment on widely available mobile devices. A pilot study was conducted to assess the feasibility, acceptability, and early effectiveness of the Sense2Quit app as a tool for individuals with HIV who are motivated to quit smoking. Upon enrollment, participants received Android smartwatches with the Sense2Quit app preinstalled. Some participants were provided with Android smartphones, while others had the Sense2Quit app installed on their own devices with assistance from the research staff. Both smartwatch and smartphone apps were used throughout this study. The pilot study lasted from March 2023 to January 2024 and involved 60 participants [21,22]. The development of the Sense2Quit app was guided by the Information Systems Research framework, incorporating focus groups, design sessions [3], and usability testing [23]. This comprehensive framework ensures a user-centered and iterative development process, incorporating valuable feedback from focus groups and design sessions with the target population, specifically people with HIV. By tailoring the intervention to this high-risk group, we aim to enhance the app’s efficacy and address the unique challenges faced by people with HIV in their smoking cessation journey. A key distinguishing feature of the Sense2Quit app is its innovative integration of smartwatches with a smartphone app (Figure 1), enabling the provision of real-time, just-in-time cessation interventions. This technology-driven approach leverages the potential of continuous health monitoring through smartwatches using a sophisticated artificial intelligence (AI) model for accurate smoking gesture detection. This paper outlines the comprehensive technical implementation of the Sense2Quit app, designed for smoking detection. This study’s core is the confounding resilient smoking (CRS) model, an AI solution specifically proposed to address the challenges of smoking detection. The CRS model is developed to enhance resilience against confounding gestures by significantly reducing false positives and negatives. This study emphasizes the importance of incorporating confounding gesture data into the training process, demonstrating how this approach improves model performance and applicability in real-world settings. Notably, the CRS model was evaluated for a high-risk population, people living with HIV, showcasing its effectiveness in addressing smoking detection in populations with unique behavioral and health care needs. The CRS model is designed for seamless integration into real-world environments with the Sense2Quit app, using standard smartwatches and smartphones with cross-platform functionality for Android and iOS systems. Additionally, we compare the CRS model against 2 state-of-the-art baselines, demonstrating its superior performance in both smoking and nonsmoking gesture detection. Generalizability experiments using leave-one-subject-out evaluation further validate the model’s robustness across diverse individuals, ensuring its reliability even for unseen participants. Recommendations are provided for adapting the CRS model to more extensive and diverse datasets, highlighting its scalability and applicability in various scenarios. Furthermore, we evaluated the smartwatch’s power consumption to ensure its continuous-use feasibility. The usability testing of the Sense2Quit smartphone app was performed to assess user experience (UX) and identify potential improvements for real-world deployment. Data collection methodologies are meticulously designed to capture confounding gesture data, with a dedicated app module developed for seamless data acquisition.

The subsequent sections of this paper are organized as follows: first, we introduce the technical components that constitute the Sense2Quit app and provide an in-depth overview of the CRS model. Next, we elaborate on the methodologies used to enhance the model’s resilience against confounding gestures, including details of the data collection protocol, evaluation procedures, and cross-platform app functionality. Following this, we comprehensively compare the CRS model with state-of-the-art baselines, discuss generalizability experiments using leave-one-subject-out evaluation, and provide recommendations for scaling the model to larger datasets. Additionally, we report on the results of smartwatch power consumption testing and Android app usability testing. Finally, we conclude with the results of our study and explore potential future directions for this research, emphasizing the CRS model’s readiness for deployment in real-world applications.

### Sense2Quit App Overview

#### Overview

Sense2Quit consists of 2 primary components: the smartphone and smartwatch apps, and an online dashboard. These elements synergistically work together to provide users with a comprehensive solution for smoking cessation, including real-time participant data tracking to address potential issues and minimize participant attrition proactively. The pilot study used Android smartphones and smartwatches, restricting participant recruitment to Android users. To overcome this limitation, we developed a cross-platform app version using Flutter, making it available for iOS and Android devices. This ensures smooth installation from both Google and Apple app stores, improving access to potential participants and enhancing user accessibility. The following sections provide a detailed overview of each component’s technical functionalities and contributions, illustrating how they support users on their journey to quit smoking.

#### Smartwatch App Development

The Sense2Quit smartwatch app serves as a crucial instrument for collecting essential movement data, thereby enabling the detection of smoking behaviors in users. Strategically placed on the participant’s wrist, this app leverages the advanced capabilities of built-in smartwatch sensors, including the 3-axis accelerometer and gyroscope, to capture intricate motion patterns. With a sampling rate of approximately 20 Hz, the smartwatch records hand movement data in real-time. This sensor data is efficiently transmitted to the user’s smartphone for comprehensive analysis. In addition to monitoring movement data, the smartwatch app offers valuable cessation tips to aid users in their journey to quit smoking. Importantly, this app is compatible with a broad range of smartwatches, encompassing Android and Apple devices. Extensive testing has been conducted on popular models such as the Ticwatch (HK SMARTMV LIMITED), Fossil watch, and Apple Watch SE, ensuring reliable performance and compatibility across various wearable platforms.

#### Smartphone App Development

**Figure 2:**
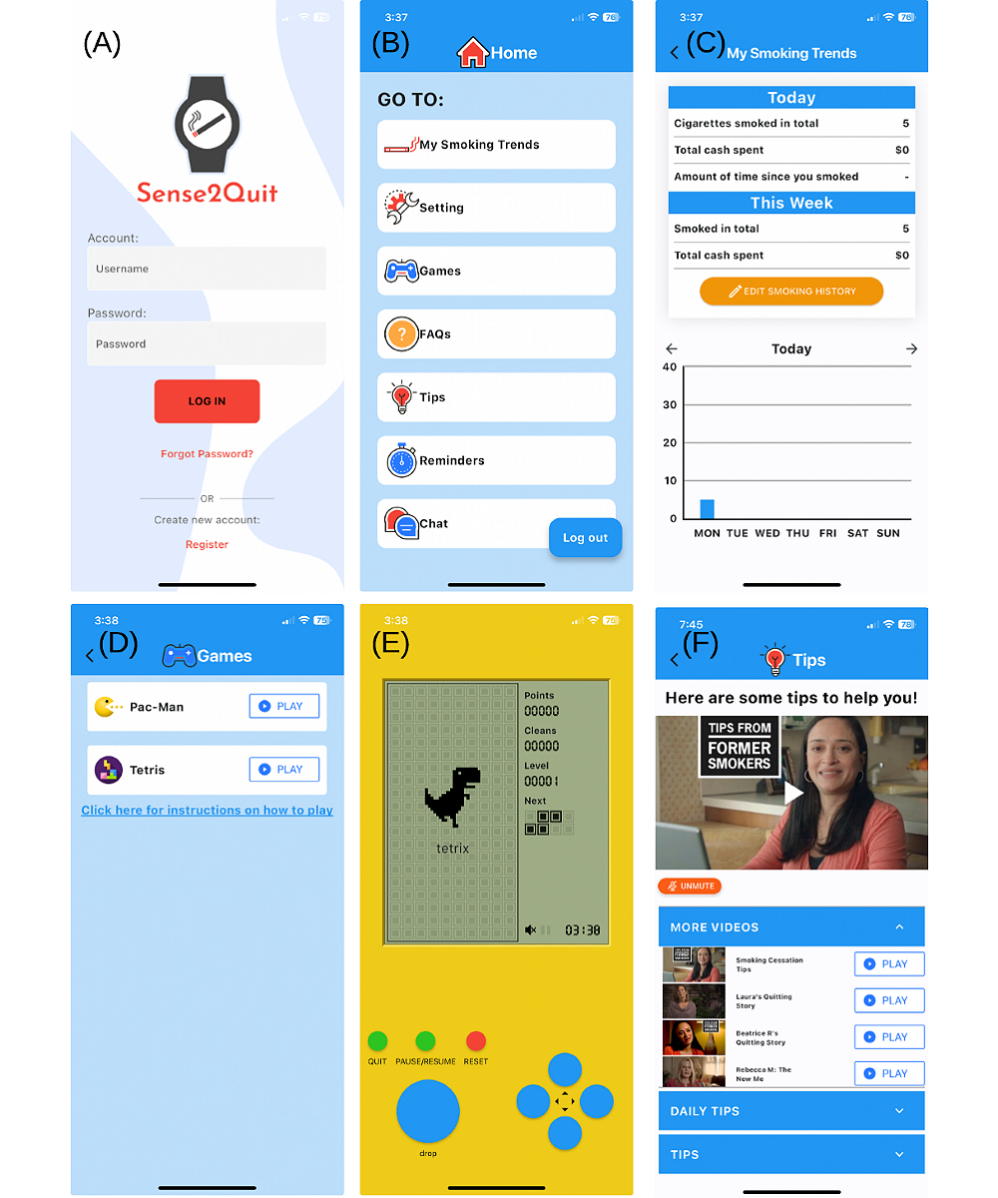
User interface screenshots of the Sense2Quit smartphone app, showcasing the (A) login, (B) home, (C) smoking trends, (D) list of games, (E) Tetris game, and (F) tips screens.

The Sense2Quit smartphone app serves as the central interface for users, offering comprehensive features to support smoking cessation (Figure 2). The app’s smoking dashboard provides a detailed summary of smoking habits, including the number of cigarettes consumed, expenditure, and daily trends, all visualized through intuitive graphs. This functionality enables users to monitor their progress and make informed decisions effectively. To help manage cravings, the app includes interactive games such as Pac-Man and Tetris, which serve as distractions to promote healthier coping mechanisms. Additionally, the tips section provides informative videos and practical advice to help users overcome challenges. The Sense2Quit app empowers users to achieve a smoke-free lifestyle by integrating monitoring, distraction, and educational components. Our earlier work details the various user interface (UI) features and usability testing [23].

**Figure 3:**
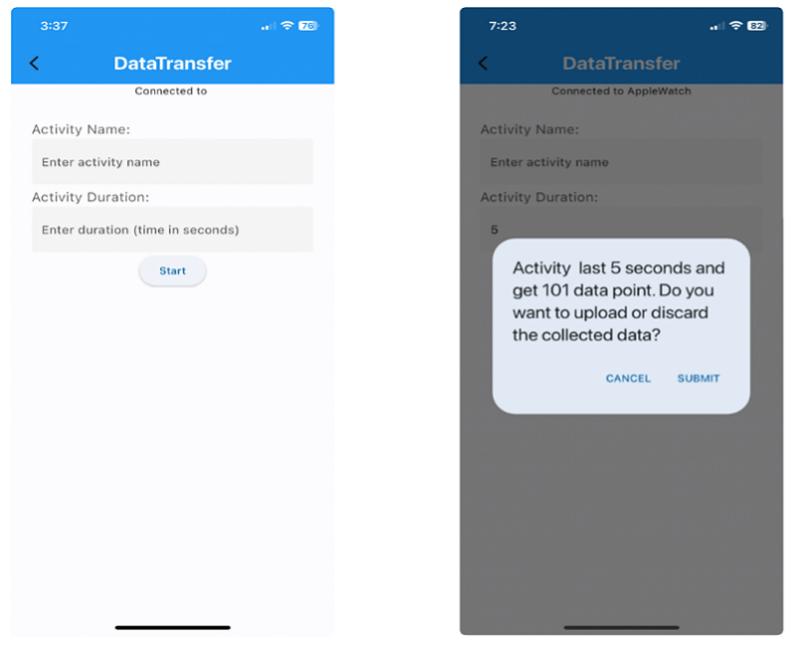
User interface for the data collection screen demonstrating the states before data collection on the left and after data collection is completed on the right.

A specialized data collection module was integrated into the app to facilitate data collection on confounding gestures. This module was designed to provide research staff with a streamlined system for gathering and uploading data to a cloud server, enabling the technical team to access and refine the baseline smoking detection model. The UI (Figure 3) was engineered for efficiency and simplicity, allowing the collection and upload of gesture data from each participant to be minimally complex. A crucial UI feature was the prominent connection status indicator positioned at the top of the screen. This indicator dynamically displayed the pairing status between the smartwatch and smartphone. When successfully connected, it showed “connected” and instantly switched to “disconnected” if the connection was lost. Significantly, any ongoing data collection would automatically halt in the event of a disconnection. This real-time feedback mechanism ensured data integrity and continuity throughout the experiment. It allowed research staff to immediately identify and address connectivity issues, thereby minimizing data loss and reducing the need for repeated trials.

The UI also included (1) a connection status indicator at the top of the screen; (2) an input field for activity names, where the gesture was performed, and the participant’s username was entered; (3) a duration input field to specify the recording length in seconds; (4) a start button to initiate data collection; (5) a countdown display indicating the remaining time for data collection; (6) a confirmation dialog postcollection to verify data quality before upload; and (7) separate indicators for data saving and successful upload to provide real-time feedback to data collectors.

The confirmation mechanism was implemented to prevent the upload of data from instances where participants could not perform gestures as intended, thereby mitigating data corruption at the source. Upon confirmation, the data was transmitted to Amazon Web Services (AWS) cloud storage, specifically to a DynamoDB instance. Each entry in the database included 3-axis accelerometer and gyroscope data, along with the activity name, participant ID, and timestamp.

The Sense2Quit app, designed for iOS and Android platforms, was developed using Flutter [24]. This cross-platform compatibility significantly improved over the previous version, which only supported Android devices, thereby restricting the inclusion of participants who used Apple devices in this study. Porting the existing Android app to a cross-platform framework presented challenges, particularly regarding the core functionality of transferring sensor data between the smartwatch and the mobile device across both platforms. The standard open-source packages in Flutter were insufficient to achieve this functionality due to platform-specific limitations. We encountered restrictions related to how long the app could run in the background and the number of background operations permitted by the package. As a result, platform-specific code had to be implemented for Android and iOS. However, the UI code remained broadly consistent across platforms, ensuring a similar UX for participants regardless of their device.

#### Connectivity

The smartwatch’s accelerometer and gyroscope sensor data are transmitted to the smartphone via a Bluetooth Low Energy connection. Establishing this connection involves a 3-way handshake, such as the TCP protocol [25]. The handshake begins with the smartphone sending a greeting message, to which the smartwatch responds with an acknowledgment message. Subsequently, the smartphone sends a “heartbeat” message to prompt the smartwatch to start transmitting sensor data. Once a participant is onboarded and the connection between the devices is established, data transfer occurs continuously in the background.

The heartbeat mechanism maintains a stable connection and uninterrupted data flow. The smartphone sends a heartbeat every 3 seconds to ensure the smartwatch continues to send data. If the smartwatch does not receive a heartbeat for 6 seconds, the data transfer ceases, and the Sense2Quit app notifies the participant via the smartphone. This method ensures reliable data transmission between the smartwatch and the smartphone.

#### Automatic Smoking Detection

**Figure 4:**
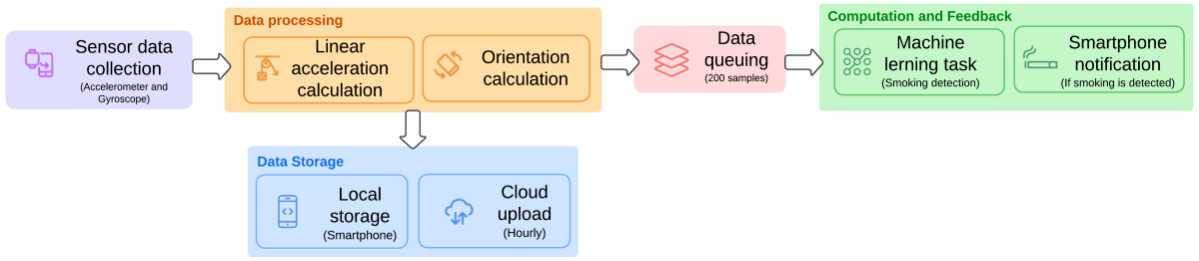
Workflow of the automatic smoking detection system, from sensor data collection and processing to using the smoking detection model to provide user feedback.

The smoking detection algorithm is divided into multiple steps (Figure 4). The sensor data is sent from the smartwatch to the smartphone. The sensor data contains a timestamp, 3-axis total accelerometer, and 3-axis gyroscope values. Gravity *g* always influences the measured acceleration; therefore, to accurately measure the actual acceleration, the contribution of gravitational force must be eliminated. This is typically achieved through filtering the data using either a high-pass or a low-pass filter. Our app implemented a low-pass filter that smooths out rapid fluctuations in the acceleration data, isolating the gravity component. The 3-axis accelerometer values sent to the smartphone are the total accelerometer values; the gravity component is separated using the following recursive low-pass filter formula:



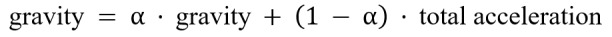



Where α is a constant between 0 and 1 that determines the responsiveness of the filter, which is set to 0.8 in our app, this is performed for all the 3 readings (x, y, z) axes. Once the gravity component is separated, the linear acceleration is calculated using the equation:



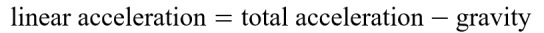



The linear equation is also performed for all 3 readings, giving us the linear acceleration values without the gravity component. The smartwatch’s gyroscope data is sent as a rotation vector representing the orientation; no further filtering is applied.

Once the data is cleaned, the smartphone stores the linear acceleration and gyroscope values locally. It then schedules the local data to be uploaded to AWS cloud storage every hour. The data is transferred from local storage to 2 queues, each holding the values until they accumulate 200 entries. These values are then processed by the machine learning model for inference, which calculates a prediction to determine if a smoking gesture occurred within the sample period. This process is repeated continuously until a smoking gesture is detected. A notification is sent upon detection, and the smoking data is recorded in the app. To avoid redundancy, no further messages are sent for 450 seconds, which is assumed to be the average duration of smoking for this application and treated as a cooldown period for smoking detection.

#### Smoking Gesture Detection Model

This study used a convolutional neural network (CNN) to enhance the accuracy of detecting smoking gestures from wearable sensor data. The CNN model was trained to classify smoking versus nonsmoking gestures using time-series data from a smartwatch’s accelerometer and gyroscope. The choice of a CNN architecture was driven by its ability to effectively extract spatial features from raw sensor data, a critical factor in differentiating smoking gestures from similar activities. Additionally, CNNs demonstrate strong performance in recognizing smoking gestures even when performed simultaneously with other actions [26], achieving high accuracy in person-independent evaluations. By integrating data from accelerometers and gyroscopes, the model’s accuracy in gesture detection improves further [27]. The CNN’s low computational complexity and high classification accuracy make it well-suited for real-time deployment on wearable devices [28].

**Figure 5:**
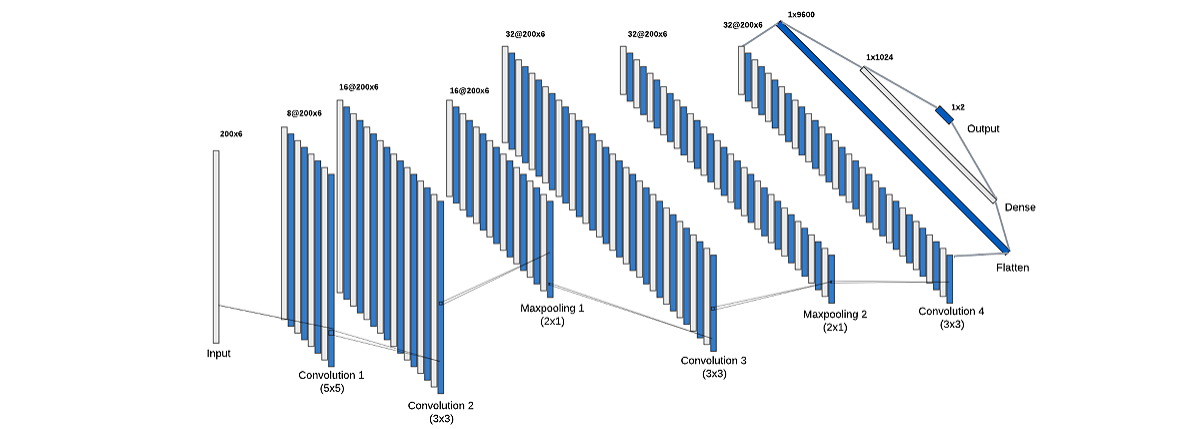
Smoking detection model architecture illustrating the underlying layers.

The input data comprised 200 time points with 6 sensor values (a 3-axis accelerometer and a 3-axis gyroscope). The architecture (Figure 5) is well-suited for this app because the input data consists of sequential sensor readings with subtle but discriminative temporal and spatial patterns. The convolutional layers enable the model to capture these patterns efficiently, while the progressively increasing filter counts (8, 16, and 32) ensure hierarchical learning of features, from simple motion patterns in the lower layers to more complex and specific smoking gestures in the higher layers. The initial larger kernel size (5×5) captures broader motion characteristics across time, while the smaller kernels (3×3) in subsequent layers refine these patterns to focus on gesture-specific features. Leaky ReLU activation is used in all convolutional layers to address the dying ReLU [29] problem and enhance gradient flow. Two MaxPooling layers (2×1) are interspersed to reduce dimensionality. The network also includes a dense layer with 1024 units and a 0.5 dropout rate for overfitting prevention. The output layer uses softmax activation for binary classification between smoking and nonsmoking gestures. This architecture is specifically designed to capture the subtle temporal and spatial patterns characteristic of smoking gestures, with its progressive feature extraction and dimension reduction facilitating efficient and accurate classification. Using Leaky ReLU, strategic pooling, and dropout contributes to robust learning and generalization, making the model well-suited for real-time smoking gesture detection in wearable devices. The categorical cross-entropy loss function, the learning rate 0.001, a total of 100 epochs, and 32 samples per batch were used for training. The model captures complementary motion information by leveraging accelerometer and gyroscope data, improving classification accuracy for smoking versus nonsmoking gestures.

#### Online Dashboard

**Figure 6:**
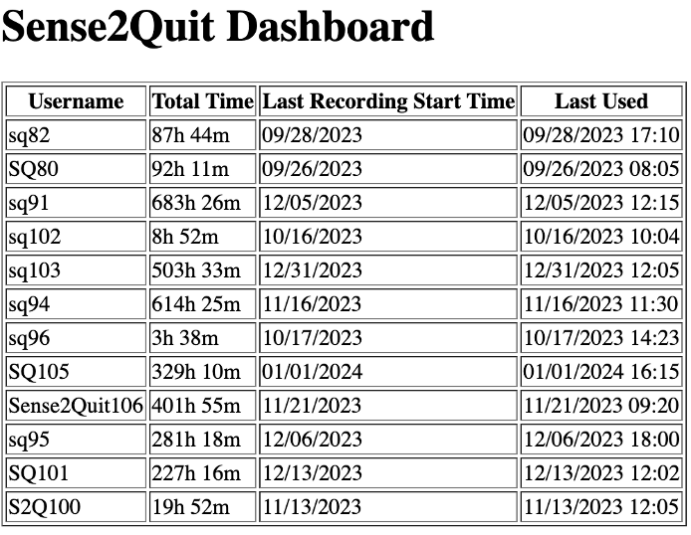
The Sense2Quit dashboard for research staff provided usage information to participants enrolled in this study.

An online dashboard (Figure 6) was developed and implemented for the research staff to facilitate real-time monitoring of study participants. This web-based interface, created using the Flask framework and hosted on AWS Elastic Cloud, provided critical insights into participant engagement and data collection processes. The dashboard displayed vital metrics for each enrolled participant, including (1) total duration since onboarding with the app, (2) timestamp of the most recent local data backup to the cloud, and (3) timestamp of the participant’s last interaction with the app.

This live information served as a vital data point for this study, enabling researchers to promptly identify any anomalies in app performance, monitor participant adherence to this study’s protocol, and initiate timely follow-ups with participants who demonstrated prolonged periods of inactivity.

## Methods

### Study Design

The pilot study revealed a significant challenge: frequent false positive notifications triggered by participants’ routine activities [21,22]. Upon investigation, we identified a range of confounding gestures and actions that closely resemble smoking-related motions, compromising detection accuracy. Confounding gestures are everyday hand-to-mouth actions that involve repetitive or subtle movements, like smoking, such as bringing the hand to the mouth or face area. Specifically, these include gestures such as eating, drinking, yawning, talking with hand movements, applying chapstick, scratching the face, adjusting glasses, waving, and answering phone calls. These gestures closely mimic the smoking motion because they involve similar wrist rotations, hand positioning, and timing patterns, presenting significant challenges to automated gesture recognition systems. Accurately differentiating these actions from genuine smoking gestures is essential to avoid false positive alerts, maintain user trust, and ensure that real-time interventions are timely and appropriate.

Additionally, as the pilot study was conducted exclusively on Android devices, we could not recruit participants who used Apple devices, which limited the overall participant enrollment. Some participants also reported connectivity issues between their smartphones and smartwatches, further complicating data collection and analysis. Addressing these limitations became crucial for improving detection accuracy and system performance. To tackle these challenges, we implemented 2 methods:

First, we recruited 30 participants from the pilot study and recorded 16 common hand-to-mouth gestures from their daily activities, including the smoking gesture. The typical smoking gesture sequence—characterized by the user bringing their hand to their mouth for inhalation, lowering it to a resting position, and then repeating the motion for subsequent puffs—is not unique to smoking. Similar action patterns can be observed in other activities, such as drinking or answering a phone call, which we have included in our investigation. This data evaluated the model’s ability to distinguish smoking-related gestures from similar actions, such as drinking or answering a phone call. These gestures were collected across 3 distinct postures to ensure comprehensive coverage of potential confounding movements.

Second, to evaluate the app’s cross-platform functionality and performance, we recruited 8 additional participants who had yet to participate in the pilot study. These participants were asked to rate the smartphone app’s usability on iOS and Android platforms. We also performed a power consumption analysis on the smartwatch to assess its energy efficiency when running the app. These combined methods enhanced the model’s discriminative accuracy and addressed previous connectivity issues without introducing additional computational complexity.

### Confounding Gestures: Experiment Design and Evaluation

The experiment aimed to collect confounding gesture data from 30 participants aged 34 to 71 (mean age 59.07, SD 8.92) years who were people with HIV, smoked cigarettes, and participated in the Sense2Quit pilot study. This sample size was chosen based on similar studies in wearable gesture recognition and smoking detection, which have successfully employed 10-30 participants to capture sufficient variability in gesture performance [12,15,18,20]. Given the challenges in recruiting a specialized high-risk population, such as people living with HIV, and the fact that each participant contributes multiple data points across various gestures and postures, this sample size is justified for this exploratory study. Participants were recruited at their 12-week follow-up appointment or through phone calls following completion of this study. The gender distribution among the participants was 16 males and 14 females. Regarding ethnicity, the cohort included 2 (6.7%) White participants, 25 (83.3%) Black participants, and 6 (20%) Hispanic or Latino participants. The participants’ mean height was 166.88 (SD 8.83) cm, their mean weight was 77.14 (SD 16.34) kg, and their mean BMI was 27.91 (SD 6.54) kg/m² (Table 1). The visits took place at Columbia University School of Nursing and lasted between 15 and 30 minutes. During the visits, participants completed a series of tasks while wearing a smartwatch on their wrists under the instruction of a research staff member. The smartwatch was connected to the Sense2Quit smartphone app, which recorded accelerometer and gyroscope data of the gestures performed. This study included 16 distinct gestures, comprising 15 confounding gestures and 1 smoking-related gesture. The list of activities that the participants performed during confounding gestures data collection is drinking without straw, drinking with straw, eating with fork, eating without fork, talking with hand gesture, using a phone (making a phone call), adjusting glasses, arm cross, scratching face, applying chapstick, yawning, pinching chin, wiping nose, messaging head, waving, and smoking. The gestures selected for this experiment were chosen because they closely resembled the motions involved in smoking, thereby serving as confounding gestures that an AI smoking detection model could misclassify. These confounding gestures simulate everyday actions that involve similar wrist and arm movements, such as eating, drinking, or waving, which are prone to generating false positives in smoking detection [30]. Each gesture was performed for 5 seconds, and participants executed them in 3 distinct postural conditions: seated, standing, and walking. However, it is essential to note that not all participants could perform the gestures in all 3 postural conditions due to the limited mobility of some participants. Data collection was limited to those postures in which participants reported and demonstrated comfort in performing the required gestures. This approach was adopted to ensure the ecological validity of the data while prioritizing participant comfort and safety.

**Table 1:** Characteristics of participants (N=30).

Characteristic	Values
Age (years), mean (SD)	59.07 8.92)
Female, n (%)	14 (46.7)
Male, n (%)	16 (53.3)
Black, n (%)	25 (83.3)
Hispanic or Latino, n (%)	6 (20)
White, n (%)	2 (6.7)
Height (cm), mean (SD)	166.88 (8.83)
Weight (kg), mean (SD)	77.14 (16.34)
BMI (kg/m^2^), mean (SD)	27.91 (6.54)

To develop the CRS model, we trained a CNN, as described in the previous section, by incorporating newly acquired confounding gesture data. The CRS model was evaluated to systematically assess the impact of confounding gestures and compare its performance against baseline and state-of-the-art models. Baseline CNN, trained without confounding gesture data, was compared to the CRS model to determine whether including such data improved resilience to false positives and negatives without altering the architecture or adding complexity. The baseline CNN had performed well in identifying smoking gestures during the pilot study but struggled with categorizing nonsmoking gestures, often misclassifying them as smoking gestures. This limitation highlighted the need to include confounding gesture data in training to address these misclassifications. The CRS model was further benchmarked against 2 state-of-the-art models. Baseline 1: a bottom-up method for monitoring smoking behavior using wrist-mounted inertial sensors [12], and baseline 2: PuffConv [11]. All models were evaluated using the confounding gestures dataset, which included data from 21 participants. Data from 9 of the original 30 participants were excluded due to corruption during collection, leaving 1054 usable samples. These samples were partitioned into training (632/1054, 60%), testing (211/1054, 20%), and evaluation (211/1054, 20%) subsets, following standard machine learning practices. Metrics for evaluation included the confusion matrix, with true positive rate (TPR) and false positive rate (FPR) defined as:



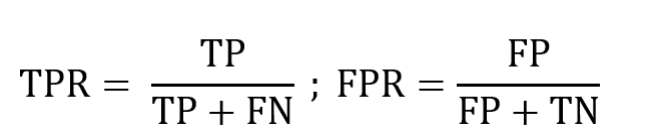



TP, FP, FN, and TN represent true positives, false positives, false negatives, and true negatives, respectively. The receiver operating characteristic (ROC) curve was used to analyze the trade-off between sensitivity (TPR) and specificity (1–FPR), with performance quantified by the area under the curve (AUC):



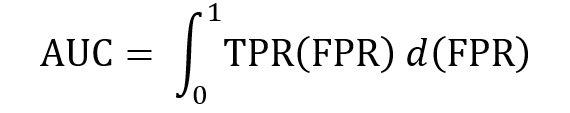



Representing the continuous summation of TPR values over the range of FPR. However, in practice, AUC is computed numerically using the trapezoidal rule based on discrete points along the ROC curve. The *F*_1_-score was also used to provide a balanced measure of precision and recall, particularly valuable for imbalanced datasets. The *F*_1_-score is defined as:



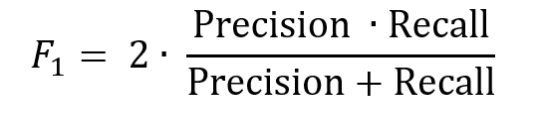



Precision (TP/[TP+FP]) quantifies the ability to avoid false positives, and recall (TPR) quantifies the ability to identify true positives. The *F*_1_-score captures the trade-off between precision and recall, ensuring a comprehensive assessment of the model’s classification performance. Finally, leave-one-subject-out cross-validation was conducted to assess generalizability. The model’s performance was evaluated on unseen participants to ensure individual robustness and applicability.

### Usability Evaluation of the Sense2Quit Apps

To assess the usability of our smartphone app on both iOS and Android platforms, a UX survey was conducted with 8 student participants from the University at Buffalo. The survey aimed to gather quantitative and qualitative data on the app’s performance, feature parity, and UI design. It was designed with questions that focused on key performance indicators such as app launch time, smoothness of the UI, and the app’s responsiveness to user inputs. Participants rated these aspects on a Likert scale [31] from 1 to 5, with 1 indicating the lowest satisfaction and 5 the highest. Moreover, the survey explored the presence of feature parity between the iOS and Android versions, probing whether any significant features available on one platform needed to be added to the other. To gather insights into the app’s overall UI design, respondents were also asked to rate the intuitiveness and usability of the app’s interface on both platforms. They provided feedback on the overall UX to identify potential improvements to enhance usability and satisfaction across different devices.

Additionally, we wanted to understand the impact of continuous data transmission on smartwatch power consumption, so we conducted a test using the Mobvoi Ticwatch Pro 3 (HK SMARTMV LIMITED), set up with an Android phone. The watch ran Sense2Quit, a custom app to collect power consumption data and the default preinstalled apps. The 20-minute test consisted of 3 phases: a 5-minute baseline phase, a 10-minute active phase with the Sense2Quit app running in the background collecting and transmitting sensor data, and another 5-minute baseline phase after the active phase. Our custom power consumption monitoring app collected current and voltage information every 5 seconds.

### Ethical Considerations

This study was conducted in full compliance with ethical standards and approved by Columbia University’s Institutional Review Board (protocol number AAAT7031). Before enrollment, all participants provided written informed consent, and every effort was made to ensure that their privacy and confidentiality were rigorously maintained throughout this study. Participants signed a consent form before participating in any study activities and were compensated with US $30 for their time.

## Results

### Confounding Gestures Dataset Evaluation

#### Overview

This section comprehensively analyzes the models’ performance on the confounding gestures dataset. First, we present the CRS model evaluation results, the baseline CNN, and state-of-the-art models, baseline 1 and baseline 2, on the collected confounding dataset. Next, we explore the CRS model’s generalizability, emphasizing its ability to perform well across diverse individuals. Finally, we examine the factors influencing individual confounding gesture performance, identifying key elements that impact the model’s accuracy and robustness for specific gestures.

#### CRS Model Performance and Comparison

**Figure 7:**
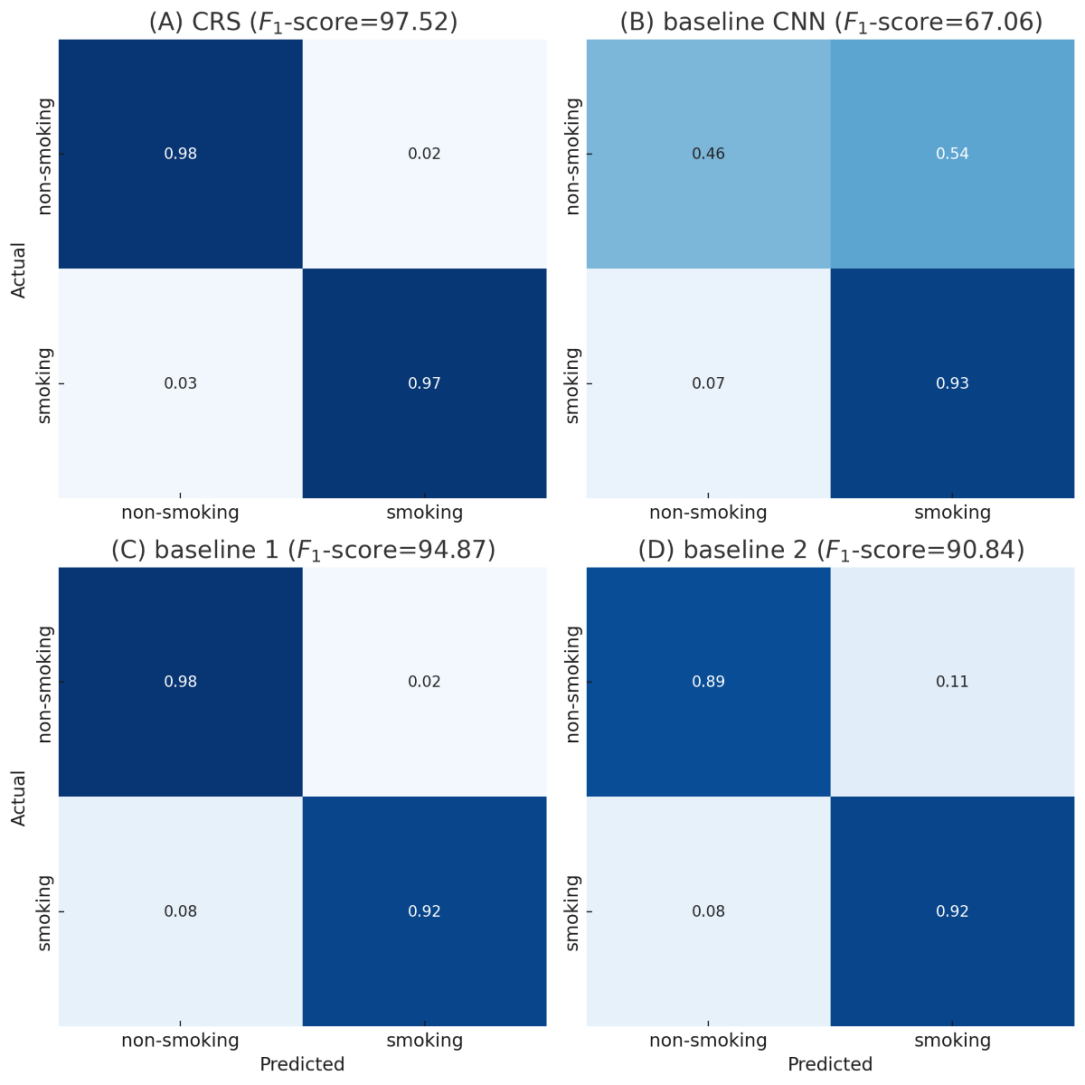
Confusion matrices for models (A) CRS, (B) baseline CNN, (C) baseline 1, and (D) baseline 2. CNN: convolutional neural network; CRS: confounding resilient smoking.

The results of our analysis underscore the substantial improvement achieved by the CRS model over the baseline CNN and the 2 state-of-the-art baselines, highlighting its effectiveness in smoking gesture recognition. The *F*_1_-score was a valuable metric for this evaluation because it combines precision (the ability to avoid false positives) and recall (the ability to identify true positives correctly) into a single balanced measure. In the context of smoking gesture detection, this balance is crucial. High precision ensures that nonsmoking gestures, such as eating or drinking, are not misclassified as smoking, thereby reducing unnecessary alerts and maintaining user trust. Meanwhile, high recall ensures that actual smoking gestures are accurately identified, minimizing the risk of missed detections and allowing timely interventions. This makes the *F*_1_-score an essential metric for evaluating smoking detection models’ reliability and practical applicability, where false positives and negatives can have significant implications for real-world use. The CRS model achieved an *F*_1_-score of 97.52, demonstrating a substantial advancement over the baseline CNN, which had an *F*_1_-score of only 67.06. The large gap between these 2 models highlights the critical role of incorporating confounding gesture data in reducing false positives and negatives, making the CRS model far more reliable and robust. Baseline CNN struggled to distinguish nonsmoking gestures, resulting in a high FPR and lower overall precision (Figure 7, confusion matrix B). In contrast, the CRS model effectively addressed these limitations, reflected in its significantly higher *F*_1_-score (Figure 7, confusion matrix A). Compared to the state-of-the-art baselines, the CRS model outperformed baseline 1 with an *F*_1_-score of 94.87 (Figure 7, confusion matrix C) and baseline 2 with an *F*_1_-score of 90.84 (Figure 7, confusion matrix D). The higher *F*_1_-score of the CRS model indicates its superior ability to balance precision (avoiding false positives) and recall (identifying true positives), even in challenging scenarios with confounding gestures. Although relatively strong performers, the state-of-the-art baselines exhibited slightly higher false positive and false negative rates than the CRS model, which could lead to occasional misclassifications in real-world applications. The CRS model demonstrated clear superiority over baseline 1 and 2 in handling confounding gesture-based smoking detection, mainly due to architectural differences. Baseline 1, which used a sequential convolution long short-term memory architecture, relied on 1D convolutional layers to extract temporal features. However, this reliance limited its ability to capture complex, hierarchical features necessary to distinguish confounding gestures from smoking gestures, particularly in scenarios with overlapping motion patterns. Similarly, baseline 2, which incorporated squeeze-and-excitation blocks within a 2D convolutional framework, endured the relatively shallow depth of its network. This hindered its capacity to extract and generalize the subtle patterns characteristic of confounding gestures, reducing its ability to separate smoking gestures from nonsmoking ones in diverse datasets effectively.

**Figure 8:**
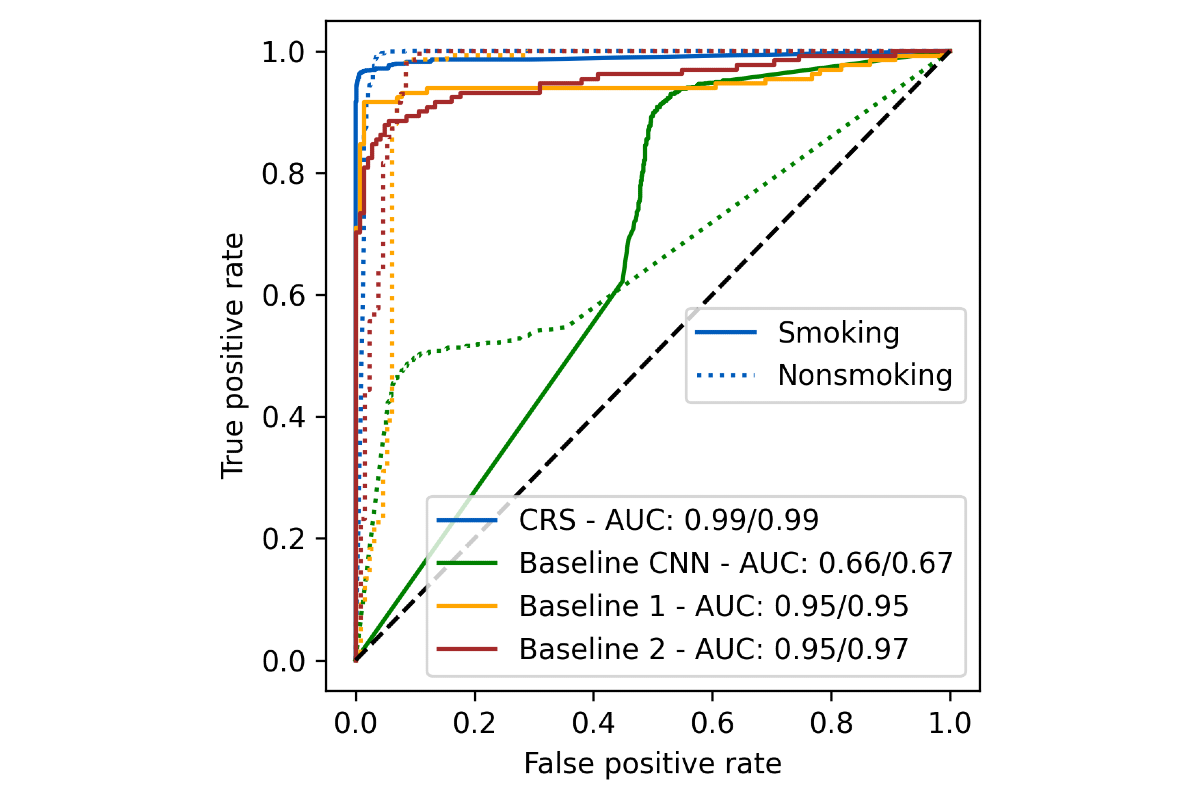
ROC curves for the smoking detection model. The blue lines represent the CRS model, the green lines correspond to the Baseline CNN, and the yellow and red lines represent the state-of-the-art baseline 1 and 2 models, respectively. Solid lines represent smoking detection, and dotted lines represent nonsmoking detection. The AUC fractions indicate the performance for smoking (first value) and nonsmoking (second value) detection. AUC: area under the curve; CNN: convolutional neural network; CRS: confounding resilient smoking; ROC: receiver operating characteristic.

We also used ROC curve analysis to visualize the trade-off between TPR (sensitivity) and FPR (Figure 8). This approach adds another layer of insight into the models’ performance. The ROC curve allows a nuanced understanding of the balance between correctly identifying smoking gestures (sensitivity) and minimizing false alarms (FPR). By analyzing the curve, we can identify an optimal classification threshold, which is especially important in real-world applications where the consequences of misclassification are significant. For instance, overly high sensitivity may increase false positives and unnecessary interventions, while low sensitivity might result in missed detections, undermining the system’s utility. The AUC derived from the ROC analysis is a robust summary of each model’s discriminative power. The CRS model achieved an impressive AUC of 0.99 for both smoking and nonsmoking gestures, significantly outperforming the baseline CNN, which had AUC values of only 0.66 and 0.67, respectively. The significant performance gap underscores the critical importance of incorporating confounding gesture data, dramatically enhancing the model’s robustness and applicability. Compared to state-of-the-art models, the CRS model still demonstrated superior performance. Baseline 1 achieved AUC values of 0.95 for both smoking and nonsmoking gestures, while baseline 2 achieved AUC values of 0.95 and 0.97 for smoking and nonsmoking gestures, respectively. While these results indicate that the state-of-the-art models are relatively strong performers, their slightly lower AUC values suggest they are less effective in distinguishing between smoking and nonsmoking gestures across various threshold settings. The state-of-the-art models’ reliance on less optimized architectures for handling confounding gestures likely contributed to this performance gap.

#### Generalizability of the CRS Model

Leave-one-subject-out cross-validation was conducted on the CRS model to evaluate its generalizability and per-participant performance. This method ensures the model’s robustness by testing its ability to accurately predict outcomes for unseen individuals, a critical requirement for real-world applications. Among the participants, SQ86 achieved the highest accuracy at 99%, indicating that the model effectively captured patterns specific to this individual’s gestures. Conversely, SQ70 had the lowest accuracy at 77%, suggesting more significant variability or overlap between smoking and nonsmoking gestures for this participant, potentially due to unique behavioral nuances or differences in sensor placement.

The average accuracy across all participants was 86%, lower than the overall *F*_1_-score evaluated across the entire dataset. This discrepancy arises because the *F*_1_-score is calculated based on the combined data from all participants, benefiting from a larger sample size and the ability to average out interindividual variability. In contrast, the leave-one-subject-out approach evaluates performance on a per-participant basis, where the model is trained without access to data from the test participant. This setup inherently amplifies the impact of interindividual differences, such as variations in gesture dynamics, sensor orientation, or individual motion patterns. However, despite the decrease in average accuracy, the result still demonstrates the model’s substantial potential for generalization. An 86% average accuracy indicates that the CRS model maintains a high level of performance even when applied to new individuals, highlighting its practical applicability and suitability for real-world scenarios where data from unseen users is inevitable. Another interesting discovery based on this analysis was that all the outliers, defined as participants with accuracy greater or less than 1 SD from the mean, were male by birth. Additionally, both the maximum (99% for participant SQ86) and minimum (77% for participant SQ70) accuracies were observed among male participants in the dataset.

Lastly, to adapt this architecture for more extensive and diverse datasets, modifications may include increasing the number of convolutional layers or filters to handle greater data complexity, incorporating recurrent layers such as long short-term memories or gated recurrent units to capture long-term dependencies in extended time-series data, and applying batch normalization for stable and efficient training. For larger datasets, dropout rates can be adjusted, and larger dense layers can be used to model complex interactions. Furthermore, optimization strategies such as learning rate schedulers and training with larger batch sizes would improve computational efficiency. These modifications ensure the architecture remains robust and effective when scaling up or transitioning to different datasets while retaining its suitability for real-time wearable apps.

#### Contribution of Confounding Gestures

**Figure 9:**
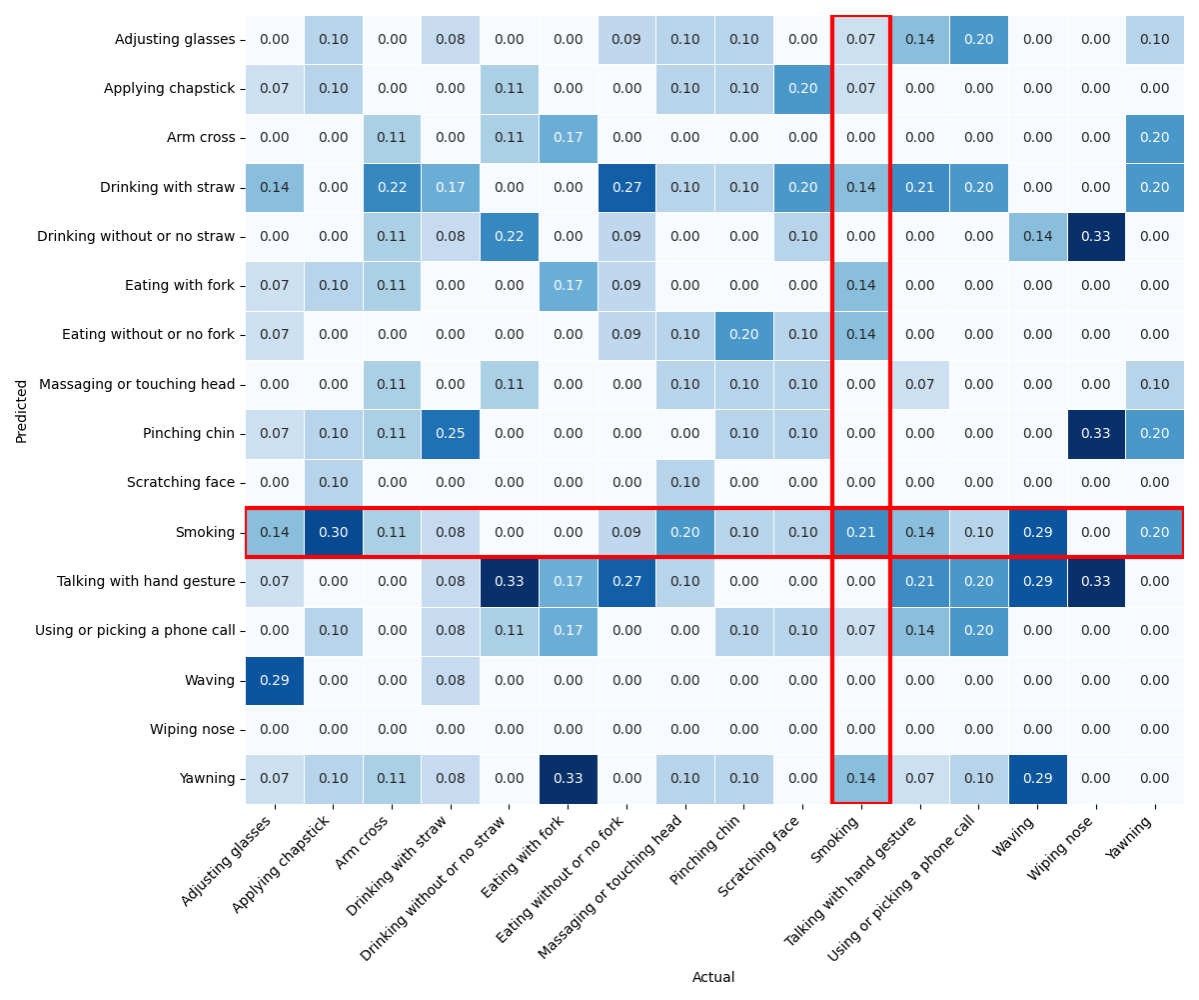
Confusion matrix for 16-class classification: red highlights indicate false negatives and false positives for the target class “smoking.”.

To better understand the confounding factors affecting gesture recognition accuracy in our model, we expanded the output layer from 2 to 16 classes. This allows us to visualize the model’s predictions in a confusion matrix (Figure 9). The matrix reveals several key insights. The actual smoking gesture was correctly predicted as smoking 21% of the time. However, the model also frequently misclassified smoking as other gestures, a type of error known as a false positive. Notably, eating with and without a fork, drinking with a straw, and yawning were incorrectly predicted as smoking 14% of the time. These gestures share similarities with smoking, such as hand-to-mouth movements, which explains the model’s confusion. Conversely, false positives occur when the model incorrectly predicts other gestures, such as smoking. The confusion matrix reveals that applying chapstick and waving were misclassified as smoking 30% and 29% of the time, respectively. These errors underscore the model’s difficulty distinguishing gestures from motion patterns such as smoking.

**Figure 10:**
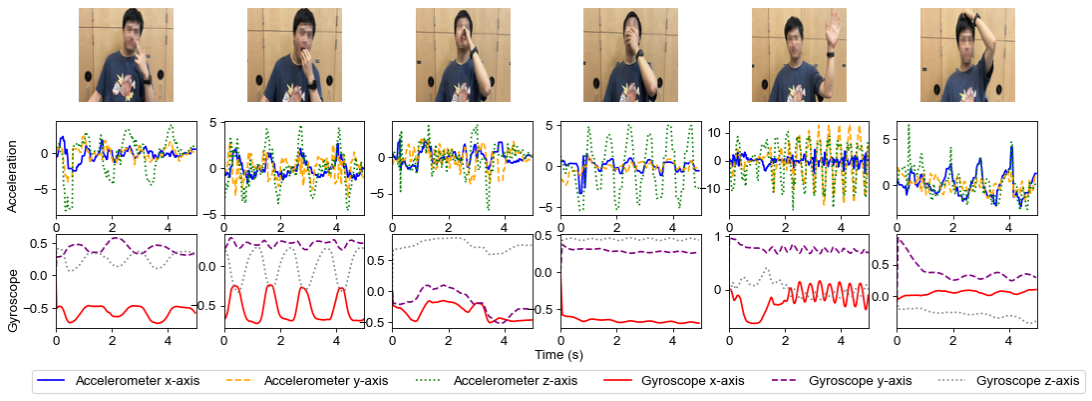
Visualization of raw accelerometer and gyroscope data of various confounding gestures (smoking, eating, drinking, yawning, waving, and scratching the head).

This pattern of errors indicates that the model’s learning process aligns with human intuition regarding gestures that resemble smoking visually or contextually. For instance, the misclassification of yawning and drinking with a straw likely stems from the model’s difficulty distinguishing between subtle hand movements near the face, daily across these gestures. The raw sensor data of these confounding gestures have been visualized (Figure 10) for better understanding. While the model effectively identifies smoking when all confounding gestures are combined, the confusion matrix highlights which specific gestures the model struggles to differentiate from smoking when each gesture is classified individually.

### Evaluation Results of the Sense2Quit Apps

The UX survey results confirmed minimal difference in usage between the Sense2Quit apps developed for iOS and Android, highlighting the app’s cross-platform functionality without negatively affecting usability (Table 2). Participants provided similar ratings across both platforms, with iOS scoring an average of 4.75 and Android scoring 4.7 for app performance. Both platforms received high scores for responsiveness, with iOS rated 4.7 and Android rated 4.8. Feature parity, however, showed a slightly lower average, with iOS scoring 4.1 and Android scoring 4.3. This difference is attributed to 2 factors: on iOS, the smoking detection feature must be manually activated by pressing the smartwatch, a restriction that stems from Apple’s watch OS, while on Android, it starts automatically. Additionally, users reported a noticeable lag in the game feature due to a bug in the Flutter package, which will be addressed in future updates. Despite these minor discrepancies, the overall UI was rated similarly on both platforms, with iOS scoring 4.7 and Android 4.5, reinforcing the app’s consistent cross-platform experience.

**Table 2:** Average Likert scores of the participants to assess the performance, feature parity, and user interface of the Sense2Quit smartphone apps.

Evaluation type			iOS score		Android score
**App performance**			4.75		4.7
	How would you rate the app launch time on the device you tested?	4.8		4.6	
	How responsive is the app to your inputs on the device you tested?	4.7		4.8	
**Feature parity**			4.1		4.3
	How well do the following features work on the device you tested: Games, adding reminders, watching tips videos, performing smoking gesture?	4.1		4.3	
**User interface**			4.7		4.5
	How would you rate the overall user experience of each device	4.7		4.5	

**Figure 11:**
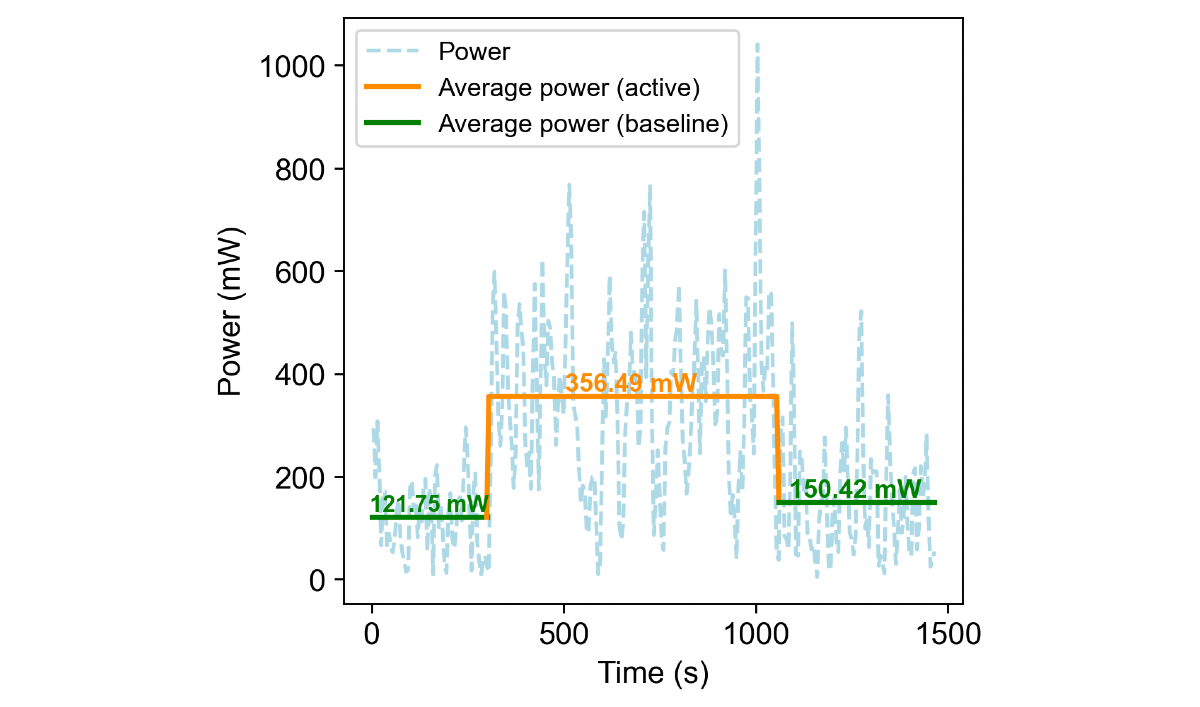
Smartwatch power consumption during active and baseline states.

The smartwatch power consumption results demonstrated that the Sense2Quit app consumed an average of 356.49 mW when active, compared to baseline averages of 121.75 mW before and 150.42 mW after the active phase. A graph (Figure 11) illustrating power consumption over time, with overlaid average power lines for baseline (green) and active (orange) phases, visualizes these results. This evaluation is crucial given that our app operates continuously in the background, potentially affecting the device’s overall power consumption and battery life. The slight increase in baseline power consumption after the active phase (from 121.75 mW to 150.42 mW) may indicate residual effects on the device’s power management.

## Discussion

This study’s primary findings indicate that the Sense2Quit system detected smoking gestures with remarkable accuracy, achieving an *F*_1_-score of 97.52% and an AUC of 0.99. This significantly improved recognition of smoking gestures compared to state-of-the-art models [11,12]. It also shows cross-platform compatibility and smooth connectivity between smartphones and smartwatches. Furthermore, user feedback indicated nearly equivalent usability on iOS and Android devices, suggesting strong potential for widespread deployment across varied mobile platforms.

The Sense2Quit study significantly advances smoking cessation technology, mainly through real-time smoking gesture recognition innovations and cross-platform app functionality. By integrating confounding gestures such as drinking, eating, yawning, and similar hand-to-mouth actions during model training, the CRS model significantly reduced false positive and false negative rates, enhancing accuracy and real-world usability. These improvements align with existing research, highlighting the critical need to address confounding activities often leading to false-positive smoking detections in wearable sensor-based interventions [32,33]. Sense2Quit provides just-in-time interventions tailored to the specific needs of high-risk populations, such as people with HIV who smoke. The system’s precise detection of smoking gestures underscores its robustness and reliability, making it an invaluable tool for smoking cessation efforts. A key strength of this study is its ability to address the long-standing challenge of confounding gestures using the CRS model, a core component of the Sense2Quit system. Integrating confounding gestures, such as drinking, eating, or other hand-to-mouth actions that closely mimic smoking, into the training process, the CRS model significantly reduces false positives and negatives, enhancing the accuracy of smoking gesture recognition. The CRS model’s ability to maintain high discriminative power while balancing sensitivity and specificity makes it particularly well-suited for real-world applications, where the consequences of misclassification can be significant. This high level of precision is achieved without adding unnecessary complexity, ensuring the model remains efficient and capable of running smoothly on mobile devices.

The inclusion of leave-one-subject-out evaluation further validated the CRS model’s robustness and generalizability, demonstrating consistent performance across diverse individuals.

Sense2Quit was developed using the Flutter framework, enabling seamless integration across Android and iOS platforms. This cross-platform compatibility significantly expands the app’s accessibility, allowing a broader user base to benefit from its real-time smoking gesture detection and intervention features, regardless of device preference. The UX of both apps was evaluated in 3 main categories: app performance, feature parity, and UI. This evaluation was based on feedback from 8 participants. The average score for iOS was 4.52, and for Android, it was 4.5. The score difference was only 0.02 on a 5-point Likert scale, indicating a similar overall UX on both platforms. The smartphone app incorporates engaging interactive elements, including tips, reminders, and games such as Pac-Man and Tetris. These help users manage cravings while keeping them actively involved with the app over extended periods. This focus on engagement through gamification enhances user retention and supports long-term smoking cessation efforts [7]. The app fosters continuous interaction by combining real-time tracking with behavioral interventions and gamification, critical for sustaining smoking cessation progress [9,10]. Regular reminders serve as behavioral prompts, reinforcing users’ focus on their quitting goals and increasing the app’s effectiveness as a daily tool in their cessation journey. The online dashboard developed for research staff provides real-time user engagement and behavior insights. This feature allows health care providers to monitor participant adherence, address potential drop-offs in engagement, and ensure adherence to this study’s protocol, ultimately improving the overall success rate of the smoking cessation program. This study also examined the power usage of a smartwatch running the Sense2Quit app and looked at how other movements could affect the accuracy of detecting smoking. These findings are essential for understanding how the app affects the smartwatch’s battery life and ensuring it remains usable for a long time. The analysis of other movements also showed that the model’s learning closely matches human intuition when distinguishing visually or contextually similar gestures, such as smoking.

While the Sense2Quit system shows great promise, areas for improvement and limitations still need to be addressed. Real-world testing is essential to validate the system’s enhanced performance in everyday situations, as the current results have been primarily obtained from controlled environments. However, insights gained from the pilot study [21,22] and the promising results of integrating confounding gestures suggest that the system will likely perform better in real-world settings. The fixed cooldown period, during which no further smoking alerts are triggered after initial detection, along with the fixed sampling rate for collecting sensor data from the smartwatch, could be dynamic to enhance detection accuracy and improve battery life. By adjusting these parameters based on real-time conditions, the system could optimize performance, reducing unnecessary power consumption while maintaining high levels of accuracy. Although the model’s ability to handle confounding gestures has significantly improved smoking detection by reducing false positives, a limitation remains in its ability to differentiate between specific nonsmoking gestures, such as eating or drinking. While distinguishing individual gestures is beyond the current scope of this study, future versions of the system could address this challenge to enhance its accuracy further. Addressing these limitations will be crucial for refining the system’s practical application and ensuring its success in real-world use. This study also revealed potential gender-related differences in the model’s accuracy, suggesting the possible influence of physiological or behavioral factors on gesture recognition. These differences may be due to variations in hand size, gesture amplitude, or smoking patterns between genders, which can affect sensor readings and model performance. To address this limitation, future research should first conduct a detailed sensor data analysis to quantify how such physiological and behavioral factors differ across genders. Specific recommendations include incorporating demographic-specific data into training and developing personalized submodels or adaptive algorithms that adjust to individual differences. For instance, using transfer learning techniques [34] to fine-tune the model on gender-specific datasets [35] or incorporating demographic variables as additional input features may help improve accuracy across diverse user groups. Moreover, collecting a larger and demographically balanced dataset would enable more rigorous statistical analysis and validation, ultimately leading to a more robust model that accounts for interindividual variability. These steps are crucial for enhancing the generalizability and equity of the system in real-world applications. Combining cutting-edge gesture recognition with user-centered design and real-time interventions, the Sense2Quit system sets a new standard for smoking cessation technology. Future research should explore integrating demographic-specific factors, real-world performance evaluations, and model optimizations to bridge the gap between advanced technology and impactful health care applications.

## Abbreviations

AI: artificial intelligence
AUC: area under the curve
AWS: Amazon Web Services
CNN: convolutional neural network
CRS: confounding resilient smoking
FN: false negative
FP: false positive
FPR: false positive rate
mHealth: mobile health
ROC: receiver operating characteristic
TN: true negative
TP: true positive
TPR: true positive rate
UI: user interface
UX: user experience
